# Encapsulation of Metal Nanoparticle Catalysts Within Mesoporous Zeolites and Their Enhanced Catalytic Performances: A Review

**DOI:** 10.3389/fchem.2018.00550

**Published:** 2018-11-09

**Authors:** Dongdong Xu, Hao Lv, Ben Liu

**Affiliations:** Jiangsu Key Laboratory of New Power Batteries, Jiangsu Collaborative Innovation Center of Biomedical Functional Materials, School of Chemistry and Materials Science, Nanjing Normal University, Nanjing, China

**Keywords:** metal nanoparticle, zeolite, mesoporous, nanoconfinement, mass transfer

## Abstract

Metal nanoparticles (NPs) exhibit desired activities in various catalytic reactions. However, the aggregation and sintering of metal NPs usually cause the loss of catalytic performance in practical reaction processes. Encapsulation of catalytically active metal NPs on/within a high-surface-area inorganic support partially resolve such concerns. Microporous zeolites, owing to their rigid frameworks and porous structural features, have been considered as one of ideal inorganic supports. Metal NPs can be easily encapsulated and stabilized within zeolitic frameworks to prevent unwished aggregation during the catalysis. Unfortunately, sole microporous nanochannels (generally <1 nm) in conventional zeolites are not easy to be accessed. The introduction of another set of nanochannel (e.g., mesopore), known as mesoporous zeolites, can greatly improve the mass-transfer efficiency, which is structurally beneficial for most catalytic reactions. The coexistence of micropores and mesopores in inorganic supports provides the synergetic advantages of both fine confinement effect for metal NPs and easy diffusion for organic reactants/intermediates/products. This review focuses on the recent advances in the design and synthesis of mesoporous zeolites-encapsulated metal NP catalysts as well as their desired catalytic performances (activity and stability) in organic reactions. We first discuss the advantages of mesoporous zeolites as the supports and present general strategies for the construction of mesoporous zeolites. Then, the preparation methods on how to encapsulate NP catalysts within both microporous and mesoporous zeolites are clearly demonstrated. Third, some recent important cases on catalytic applications are presented to verify structural advantages of mesoporous zeolite supports. Within the conclusion, the perspectives on future developments in metal NP catalysts encapsulated within mesoporous zeolites are lastly discussed.

## Introduction

Nanocatalysis by metal nanoparticles (NPs) have always attracted sufficient attention from both scientific researches and industrial applications (Astruc et al., 2005; Narayanan and El-Sayed, 2005; Astruc, 2008; Campelo et al., 2009; Cuenya, 2010). Owing to the high surface-to-volume ratio and surface energy, the surface atoms on metal NPs are supposed to be catalytically active for heterogeneous catalytic processes, such as hydrogenation, C-C coupling reaction, carbonylation, oxidation, methanol reforming, fuel cell and so on (Hartwig, 1998; Dyker, 1999; Dupont et al., 2002; Moreno-Manas and Pleixats, 2003; Na et al., 2004; Gilroy et al., 2016). However, precisely because of the high active surface atoms (unstable thermodynamic state), the aggregation and deactivation of the metal NPs by secondary nucleation and recrystallization (or Ostwald ripening) suffer the most serious drawbacks when employed in practical catalytic reactions and hence set great limits on their widely industrial applications (Schmid et al., 1996; Doyle et al., 2003; Challa et al., 2011; Schauermann et al., 2012; Hansen et al., 2013). As a result, the stabilization of metal NPs becomes a challenging research area and can be mainly realized via the combination with soft organic species and hard (or solid) supports (Astruc et al., 2005; Cao et al., 2010). The soft species, also known as capping reagents, are usually classified as polymers (including dendrimers; Corbierre et al., 2001; Crooks et al., 2001), organic or inorganic ligands (Pan et al., 2001), surfactants or micelles (Liz-Marzán and Lado-Touriño, 1996; Kitchens et al., 2003), microemulsions (Capek, 2004), ionic liquids (Dupont et al., 2002; Dupont and Scholten, 2010) and so on. By means of direct chemical binding (generally through heteroatoms) weakly to the surface of metal NPs, these soft protectors will availably block the NP aggregation but also result in the loss of active sites in the meantime due to the subsistent coverage or interfacial interaction (Jin et al., 2017). Additionally, how to resolve the essential question about the recycling potential of these capping metal NPs is always a big challenge. Another significant strategy focuses on the encapsulation of small metal NPs on some hard or solid supports, which will not only prevent NP migration and coalescence but also greatly increase the recycling accessibility (Astruc et al., 2005; Gallon et al., 2007; Campelo et al., 2009; White et al., 2009). Furthermore, the electronic properties of metal NPs on their host support environments can be greatly modified and hence will give rise to various special physical and chemical functions owing to the strong interactions between metal NPs and supports (Ju-Nam and Lead, 2008; Liu et al., 2015, 2017b). Numerous types of nanomaterials can play the role of solid supports, mainly including metal or non-metallic oxides (e.g., TiO_2_, ZrO_2_, CeO_2_, ZnO, Al_2_O_3_, Fe_3_O_4_, SiO_2_); (Astruc et al., 2005; Campelo et al., 2009), carbon (e.g., carbon sphere, nanotube, graphene, porous carbon); (Joo et al., 2001; Sun and Li, 2004), functionalized polymers (Kralik and Biffis, 2001), and porous framework (MOFs, COFs, zeolites, silica); (Dhakshinamoorthy and Garcia, 2012; Farrusseng and Tuel, 2016; Zhu and Xu, 2016). The oxide supports can classified as inert (e.g., SiO_2_) and reactive types based on their chemical reactivity. For example, CeO_2_ is a representative kind of reactive oxide support as an enhanced anchoring effect of Ce-O-M born of strong metal-support interaction can efficiently suppress the phase transformation and improve the thermal stability of metal NPs under higher temperature (Alessandro, 2002). When superparamagnetic Fe_3_O_4_ is utilized as the support, the metal NP/Fe_3_O_4_ nanomaterials can be easily recovered using a permanent magnet and therefore reused in many runs (Jacinto et al., 2008). Carbon nanostructures, owing to their intrinsic properties, such as high surface area, high electrical conductivity, and unique physical properties and so on, are extremely attractive supports and have been widely employed in the heterogeneous catalysis process (Wildgoose et al., 2006; Kamat, 2009; Liu et al., 2016, 2018). Furthermore, the carbonaceous surface can be easily modified to stabilize the metal NPs. In recent years, the combination between porous frameworks and metal NP technologies has attracted sufficient research and obtained fruitful results (Joo et al., 2001; He et al., 2003; Yang et al., 2003; Mandal et al., 2004; Rioux et al., 2005; White et al., 2009; Aijaz et al., 2012; Lu et al., 2012; Xu et al., 2012). A porous framework generally composed of interconnected network system of pores with distinct diameters, which exist in many natural substances and can be easily created in most of solid chemical nanomaterials. The pores inside the solid structures (usually called interior pore system, while exterior surface can only be accessed in non-porous structures) can nanoconfine the size and shape of metal NPs and inhibit their aggregation and further growth. The throughout pore network renders the mass-transfer more achievable during the catalysis (Satterfield, 1970; Liu et al., 2010, 2017a). Furthermore, the different components of frameworks, dimensions of porous nanochannels, pore sizes and corresponding combinations (hierarchical porosities) endow porous supports with more functional features (Davis, 2002a; Rowsell and Yaghi, 2004). Mesoporous silica with numerous pore sizes, morphologies, and mesochannels have been successfully synthesized by different strategies and also utilized as the support media of metal NPs (Kresge et al., 1992; Zhao et al., 1998; Ciesla and Schüth, 1999; Wan and Zhao, 2007). Additionally, porous carbon materials and MOFs have also been widely prepared to act as the supports for some particular catalytic applications. Each porous support possesses its intrinsic advantages (e.g., carbon receiving greater thermally stabilization) and disadvantages (for example, complex synthesis processes for mesoporous carbon, low framework stabilization of conventional mesoporous silica and MOFs); (Zhao et al., 2006; Arico et al., 2011; Slater and Cooper, 2015).

Compared to abovementioned support media, porous zeolites, due to their inherent structural features, are promised to be an important family of solid supports for the encapsulation of metal NPs. Zeolites, as a kind of highly crystalline inorganic materials having orderly distributed micropores with the diameter <1 nm, have many applications, especially in the field of petrochemical industry (Corma, 1997; Cundy and Cox, 2005; Li and Yu, 2014; Primo and Garcia, 2014; Zaarour et al., 2014). The zeolite framework commonly consists of TO_4_ tetrahedra (T denotes Si, Al, and P, etc.) which will build lots of senior structures. Up to 2018, there are totally 239 zeolite framework types (http://www.iza-structure.org/databases/) collected by the International Zeolite Association (IZA) (Guo et al., 2017). Based on different pore windows from TO_4_, conventional zeolites can be categorized into small, medium, large, and extra-large pores. The catalytic activities of zeolites largely depend on the structural and compositional features, including pore sizes, channel types and framework compositions. Compared to other catalyst supports, zeolites possess an important and unique feature, known as shape selectivity (e.g., reactants, products and transition-states). When utilized as the solid supports, the regular cavities and nanochannels in zeolites can act as the spaces and sites for the encapsulation of extraneous NPs. When metal NPs form and grow inside the micropores, they would be well-confined at the level of ultrasmall sizes and be not easy to escape from these interconnected cavities. For example, widely studied and applied zeolite ZSM-5 (MFI topology) possesses 10-numbered rings and admits the largest pore accessibility of *ca*. 0.64 nm (Kokotailo et al., 1978), which will greatly confine the crystalline growth of embedded metal NPs. Rigid framework of zeolites offers the important advantages for their applications in harsh catalytic environments; their unique nanochannels also provide the opportunity for shape-selective catalysis (Corma, 1997; Weitkamp, 2000; Davis, 2002a; Moller et al., 2012). So far, many reports on the preparation of zeolite-encapsulated metal NPs show the enhanced catalytic activities. For example, Wang et al. (2016) reported the preparation of ultrasmall Pd NPs encapsulated *in situ* within nanosized silicalite-1 by one-step hydrothermal synthesis. The resultant catalyst showed a highly efficient H_2_ generation activity toward the decomposition of formic acid. Liu et al. (2017a) prepared single Pt atoms and Pt NPs with exceptionally high thermal stability within purely siliceous MCM-22 and the catalyst presented good catalytic activity in the dehydrogenation of propane to propylene. Zhang et al. (2017) found that Pd@Beta catalyst exhibited superior selectivity for the hydrogenation of the nitro group when fixing Pd NPs inside Beta zeolite crystals. In addition, there are still many such cases in which metal NPs were well-immobilized inside specific zeolites that exhibited enhanced catalytic performances (Laursen et al., 2010; Moreno et al., 2013; Mielby et al., 2014; Xing et al., 2014; Cheng et al., 2015; Ren et al., 2015; Rubio-Marques et al., 2015; Hosseiniamoli et al., 2018; Tang et al., 2018; Yang et al., 2018).

**Figure 1:**
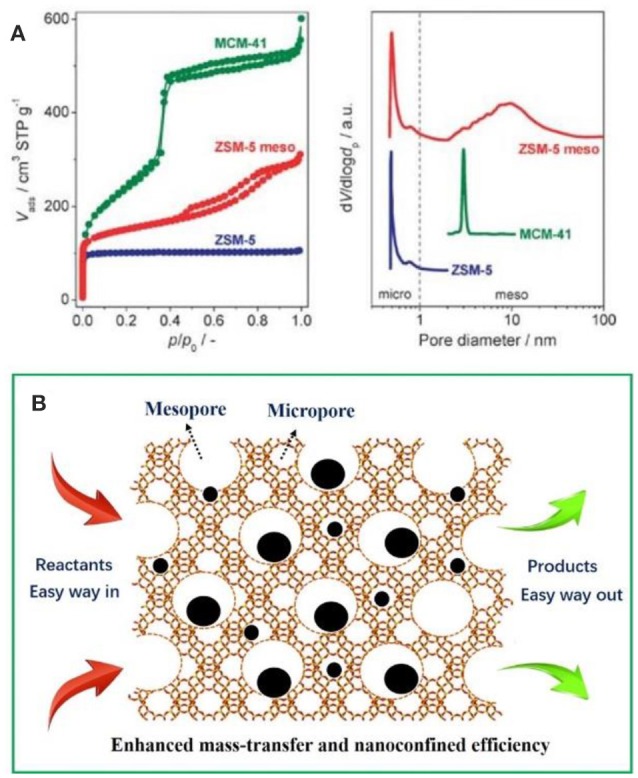
**(A)** Nitrogen isotherms and corresponded pore size distributions of characteristic porous solids with different diameter. Reprinted with permission from Perez-Ramirez et al. (2008). Copyright 2008 Royal Society of Chemistry. **(B)** Schematic representation of the advantages of mesoporous zeolites-encapsulated metal NP catalysts.

It is easy to envision that the mass-transfer processes will be greatly restricted as the micropores have been occupied by metal NPs especially involving organic molecules with larger dimensions (Kärger and Ruthven, 1992; Chen et al., 1994; Tao et al., 2006). For purpose of overcoming this shortcoming, massive efforts have been made to construct zeolitic frameworks with larger aperture (Li and Yu, 2014) or create a new set of pore system (known as mesopores) inside the conventional bulk zeolites (Egeblad et al., 2008; Perez-Ramirez et al., 2008; Lopez-Orozco et al., 2011; Möller and Bein, 2013; Parlett et al., 2013; Perego and Millini, 2013; Serrano et al., 2013). Comparatively speaking, the latter is simpler to implement. As shown in Figure 1A, purely microporous zeolites (e.g., ZSM-5), ordered mesoporous materials (e.g., MCM-41), and mesoporous zeolites (e.g., meso-ZSM-5) show obviously different Brunauer–Emmett–Teller curves and pore size distributions based N_2_ isotherms (Perez-Ramirez et al., 2008). It clearly illustrate that mesoporous zeolites possess both the micropores and mesopores (hierarchical pore systems) inside one crystal, which would be greatly helpful for the mass thransfer of organic molecules. In mesoporous zeolites-encapsulated metal NPs catalysts, metal surfaces (providing the catalytic active sites), the micropores or mesopores (immobilizing and stabilizing the metal NPs) and interconnected mesopores (accelerating the transfer of reactants and products) together built an excellent catalysis system. As schemed in Figure 1B, the metal NPs can be immobilized inside both micropores and mesopores. The reactants go through the catalysts along the interconnected mesopores and accelerate the organic reactions. The interior metal NPs also participate in the catalysis, which is not accessible in sole microporous systems.

Considering all of above aspects, this review will highlight the recent progresses on the construction of mesoporous zeolites-encapsulated metal NPs and the investigation of their enhanced activities on various catalytic applications. Conventional zeolites-encapsulated counterparts are not discussed in detail here as some excellent review papers have presented before (Farrusseng and Tuel, 2016; Wang et al., 2018b). In current review, we firstly concern design principles and construction of mesoporous zeolites. Second, we place special focus on how to immobilize or introduce metal NPs into the mesoporous zeolites. Some reported cases will be clearly presented to illustrate the synthetic process of this type of composite catalysts. Third, several typical organic reactions based on mesoporous zeolites-encapsulated metal NPs are recommended to confirm their superior catalytic performances. This review is prospected to provide some important insights on the construction of stabilized metal NPs within mesoporous zeolites and how to tune the catalytic performances based on the distinct supports media.

## Construction of mesoporous zeolites

**Figure 2:**
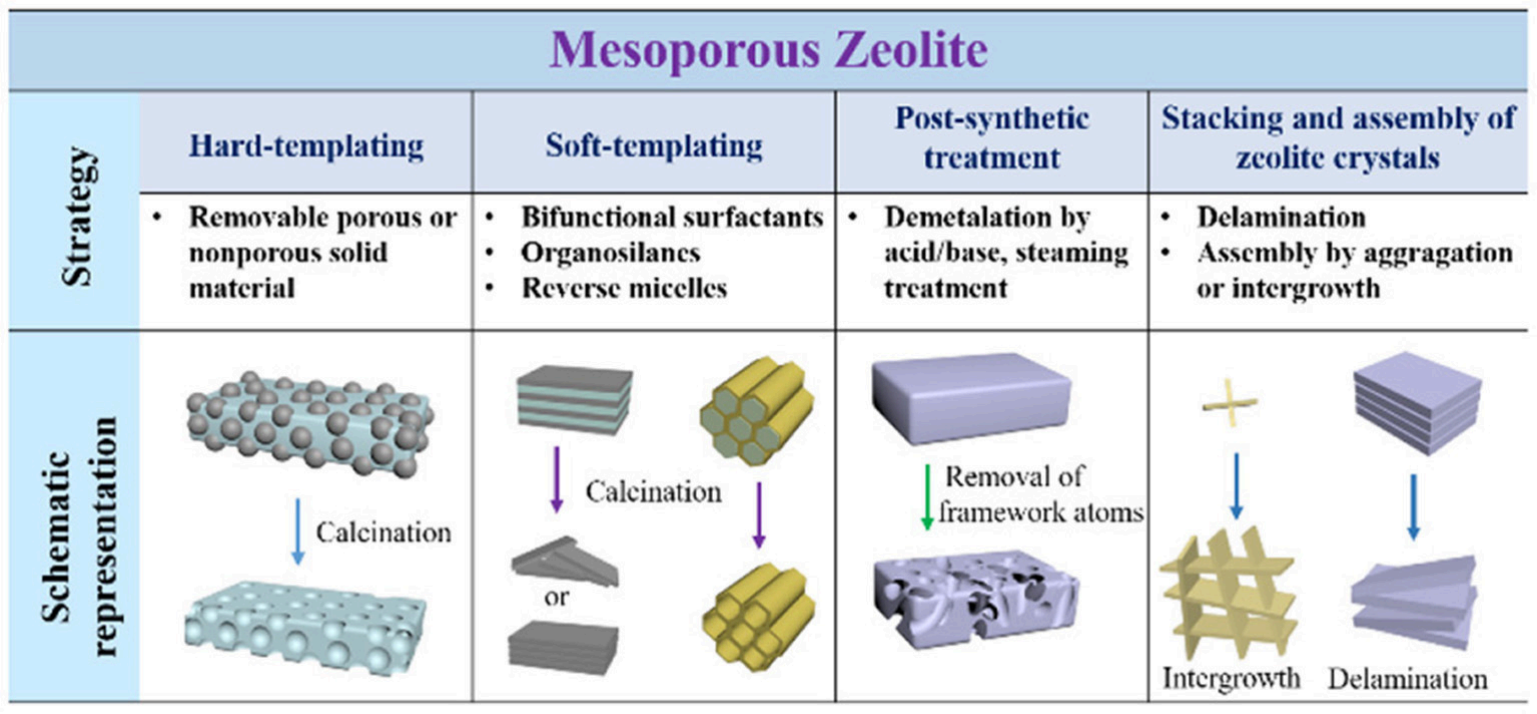
Schematic representation of the different strategies for the construction of mesoporous zeolites.

Considering the advantages of hierarchically mesoporous zeolites in various catalytic reactions, many attempts have been made to build the meospores in conventional zeolites. Furthermore, the preparation of mesoporous zeolites is the precondition for the encapsulation of metal NPs. Therefore, the synthetic strategies for mesoporous zeolites are briefly presented here (Lopez-Orozco et al., 2011; Na et al., 2013; Parlett et al., 2013; Serrano et al., 2013; Li et al., 2014a; Shi et al., 2015; Schwieger et al., 2016). The main methods can be generally classified as four aspects: (i) hard-templating, (ii) soft-templating, (iii) post-synthetic treatment, and (iv) stacking or assembly of zeolite crystals. As shown in Figure 2, the strategies and the detailed routes are clearly presented schematically.

Most of removable and inert solids with the nanoscale sizes can be employed to act the hard templates for the synthesis of mesoporous zeolites by a hard-templating method, for example, carbon nanostructures (NPs, nanowires, nanotubes, aerogel, ordered porous frameworks), CaCO_3_ NPs, polystyrene arrays, biological wood and starch (Schwieger et al., 2016). After the removal of these solid species by calcination or acid dissolution, abundant mesopores can be released while the order degree of these mesopores mainly depends on the nanostructures of hard templates. As an instance, three-dimensional ordered mesoporous carbon with large mesopores was developed to construct ordered porous zeolites with distinct framework topologies (e.g., MFI, LTA, FAU, BEA); (Chen et al., 2011). Nevertheless, although the synthesis of mesoporous zeolites using hard-templating methods is greatly high-efficiency, this strategy is often restricted due to the multistep procedures, high costs and health hazards involving during the synthesis.

Soft-templating strategy, referring to the molecular self-assembly of amphiphilic surfactants, is considered as the most possible way to build ordered mesoporous zeolites. The ordered mesopores are greatly favorable for the mass-transfer in the catalytic processes. Choi et al. (2009) and Na et al. (2011) have successfully designed a series of amphiphilic molecules containing quaternary ammoniums, also known as bifunctional surfactants, which were further employed to direct the growth of zeolitic frameworks and ordered mesoscale structures by one-step hydrothermal synthesis. As a consequence, lamellar MFI zeolite nanosheets with a single-unit-cell thickness along *b*-crystallographic axis and a hexagonally-ordered mesoporous aluminosilicate, consisted of truly crystalline zeolitic walls, have successfully obtained. By means of the introduction of aromatic groups into the hydrophobic chain, Xu et al. (2014a,b) prepared single crystalline mesostructured zeolite nanosheets and the ones with 90° rotational boundaries due to the π-π stacking between benzene rings. Additionally, Liu et al. (2012) and Zhu et al. (2014) developed the hydrophilic cationic polymer (e.g., polydiallyldimethylammonium chloride, polystyrene-*co*-4-polyvinylpyridine) to produce single-crystalline mesoporous zeolite Beta and MFI, respectively. The soft-templating method provides the possibility for the better control of both zeolitic framework topologies and mesoporous structures. However, the current synthesis is limited to zeolite MFI or Beta, and therefore how to design the effective organic surfactants for synthesizing other kinds of zeolitic topologies or mesoporous nanostructures is still a big challenge.

The selective removal or extraction of zeolite framework atoms (e.g., Si, Al, or Ti) via post-treatment by acid, alkali, steaming, or other similar methods, can produce some mesopores inside the bulk zeolites. Through the precise control of the desilication or dealumnization from the zeolite frameworks, one could obtain mesoporous zeolites with different Si/Al ratios and desired catalytic sites. This method can effectively result in abundant intracrystalline mesopores but simultaneously decrease the crystallinity of zeolites partially (Groen et al., 2005). The intercrystalline mesopores are a type of mesopores from the stacking of nanocrystals and can be easily found in most of nanomaterials. Through the synthesis of nanosized zeolite crystals, abundant mesopores although in disordered forms but may be destroyed again after the dispersion in the solvents. After the treatment like swelling, intercalation, delamination and pillaring, some layered zeolites can be turned into ultrathin zeolite layers, which further assemble into mesoporous nanostructures. A typical case is the delamination of as-synthesized MCM-22 into two-dimensional ITQ-2, which possess high surface areas and improved catalytic performances (Corma et al., 1998).

Another strategy to construct mesopores in zeolites focuses on the particular crystalline growth model of some particular zeolites. For example, through the intergrowth of MEL/MFI, self-pillared pentasil zeolite with house-of-cards-like morphology was successfully prepared. The hierarchical porosity was created inside the three-dimensional nanosheet assembly by the repetitive branching of the MFI-type zeolitic nanosheets (Zhang et al., 2012). The synthesis process based on this strategy is relatively simple (no need of special templates or tough condition) but only happens in a handful of zeolitic topologies.

Based on the discussion of the strategies to construct mesopores inside zeolites, the different synthetic routes obviously possess their intrinsic advantages and disadvantages. One should consider these processes comprehensively before the choice of a certain route and further combine the corresponded application fields. For instance, when utilized as a porous support, all of routes are suitable for the post-synthetic encapsulation of metal NPs (discussed in next part) but not for the one-step formation of mesoporous zeolite-encapsulated metal NPs composites.

## Encapsulation strategies for metal NPs

The encapsulation of metal NPs inside the porous zeolites can be mainly classified into two types of strategies: post-synthetic process and one-step *in situ* confinement. The post-synthetic strategy refers to the introduction of metal NPs after the complete construction of zeolitic framework. Contrastively, the one-step confinement method needs the co-crystallization of zeolites and metallic precursors, and follows an *in-situ* reduction to obtain metal NPs.

### Post-synthetic encapsulation strategy

**Figure 3:**
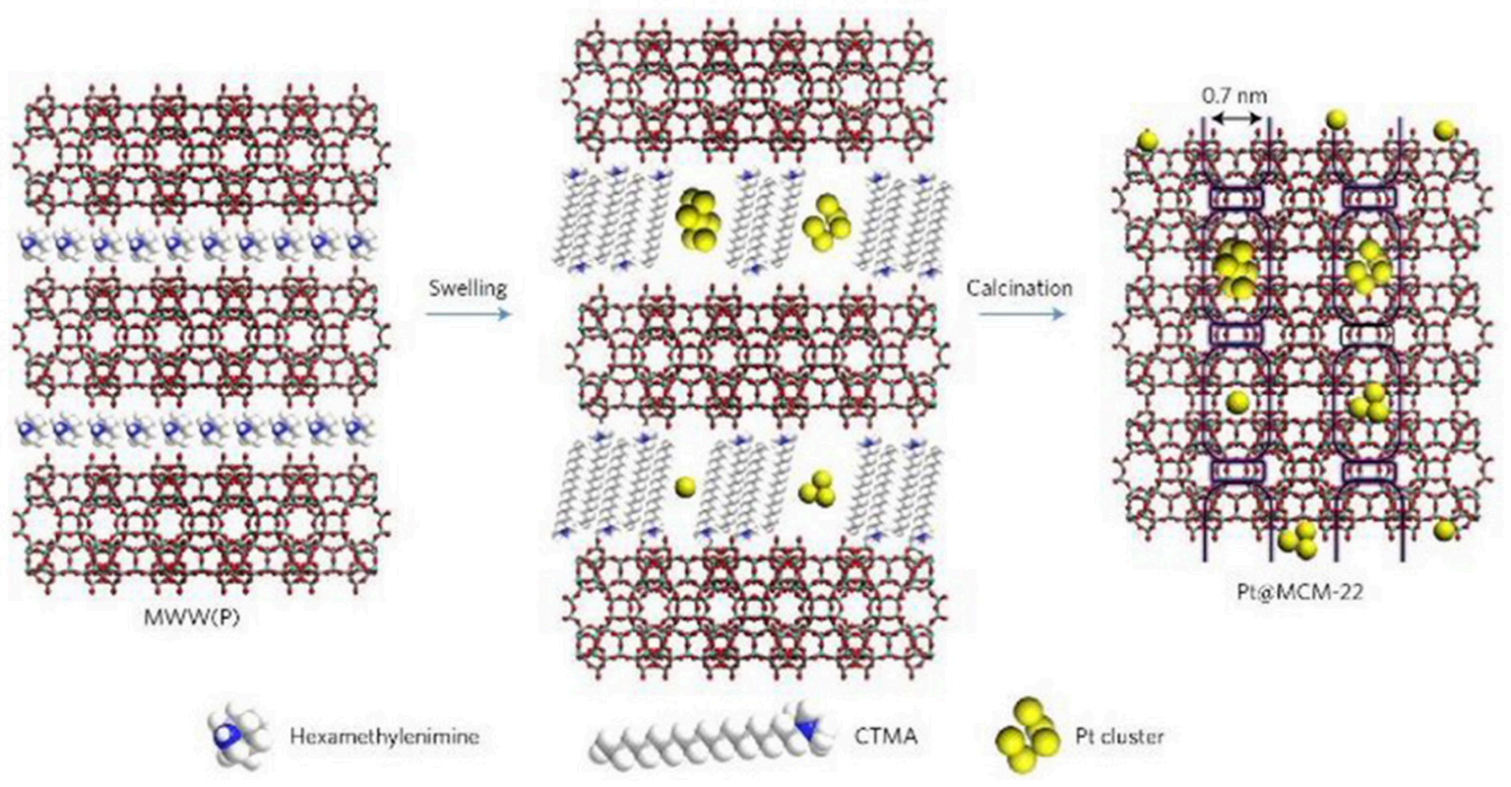
Schematics of the processes for the preparation of Pt@MCM-22. Reprinted with permission from Liu et al. (2017c). Copyright 2016 Macmillan Publishers Ltd: (Nature Materials).

The post-synthetic strategy is widely utilized to introduce metal NPs into zeolite crystals as it does not limit the framework sorts of zeolites. The process based on this strategy can be reached by means of the soak of zeolite supports in metal colloids (or NPs) or soluble metal precursors. The channels and pores inside zeolites provide the region for the loading of metal NPs. The diffusion of metal NPs or precursors is usually accelerated in a set of relatively large micropores, especially in mesopores. Actually, the diffusion of metal NPs is quite difficult to enter into the interior structures of zeolites where most of them only load at the surface (Wang et al., 2018a). As a representative example, by means of a particular structural transformation of two-dimensional (2D) zeolite into three-dimensional (3D), Liu et al. (2017c) had successfully imbedded single Pt atoms and Pt NPs inside siliceous MCM-22 (Pt@MCM-22). As shown in Figure 3, during the swelling process of the lamellar precursor MCM-22 where MWW layers were expanded by the surfactant hexadecyltrimethylammonium, subnanometric Pt species were incorporated into internal channels between individual MWW layers. After the removal of organic species by calcination, lamellar MCM-22 would turn into 3D MCM-22 and simultaneously Pt species were well-confined in external cups (hemi-cages) or encapsulated in the supercages. Subnanometric Pt species were tightly entrapped inside zeolite MCM-22. Due to structural features, Pt@MCM-22 possessed exceptionally high thermal stability even after the treatment in air up to 540°C and showed desired size-selective catalytic activity for the hydrogenation of alkenes.

Most of cases for the preparation of zeolite-encapsulated metal NP catalysts focus on the strategy using soluble metal precursors, also called wetness impregnation method. In this process, metallic species can easily diffuse into the crystal inner and load in the micropores or mesopores after the removal of solvent (e.g., water). Following a conventional reduction step, the metal precursors can be reduced into elementary metal NPs. A synthesis methodology using thiol-based organometallic complexes was presented to encapsulate metal NPs within LTA zeolites, which were successful in encapsulating 1–2 nm monometallic (e.g., Pt, Pd, Ir, Rh, and Ag) or alloyed NPs (e.g., AuPd, AuPt, and PdPt); (Choi et al., 2010; Otto et al., 2016). In pure microporous zeolites, limited by the small diameter of micropores, the size of resultant metals was usually confined in the region of below 2 nm. As for mesoporous zeolite supports, it is more easy to immobilize metal NPs owing to the presence of the larger mesopores. So far, many kinds of metal NPs (Pt, Pd, Ru, Ag, and the corresponded alloys) has been immobilized in mesoporous zeolites with different framework topologies based on this wetness impregnation method (Wang et al., 2015, 2018a; Mendes et al., 2017; Chen et al., 2018; Zhang et al., 2018). Due to the intrinsically structural advantages of mesoporous zeolites and confinement effects for the immobilized metal NPs, all of these reported composite catalysts had exhibited superior catalytic activity in various catalytic reactions (Liu and Corma, 2018).

### *In situ* confinement strategy

Metal NPs or precursors can also be introduced into the zeolite crystal inners via the one-pot hydrothermal synthetic process. In this method, as-synthesized metal NPs or soluble metallic precursors were firstly mixed with the synthetic gel for zeolites (e.g., structural-directing agents, silica resources, water, sodium hydroxide, etc.), and then turned into high temperature for the crystallization of zeolites. The as-synthesized composite products were further calcined to remove the organic species and reduced under reduction agents (e.g., H_2_, NaBH_4_) to form metal NPs. Laursen et al. reported the synthesis of zeolite-encapsulated Au NPs for size-selective satalysis (Laursen et al., 2010; Højholt et al., 2011). Ultrasmall Au NPs were uniformly immobilized in silica matrix that would be turned into a zeolite phase (silicalite-1). Au NPs embedded inside slicalite-1 mostly possessed a diameter of 1–2 nm; no obvious change in the original size can be seen even under calcination under 500°C. The crystalline zeolite frameworks played the role to confine the growth of Au NPs. However, in this process, a phase separation phenomenon often happened due to the harsh crystalline conditions for zeolites, resulting in the formation of larger metal aggregates. Therefore, the usage of metal NPs as the precursors is not widely applied for the construction of zeolite-encapsulated metal catalysts.

**Figure 4:**
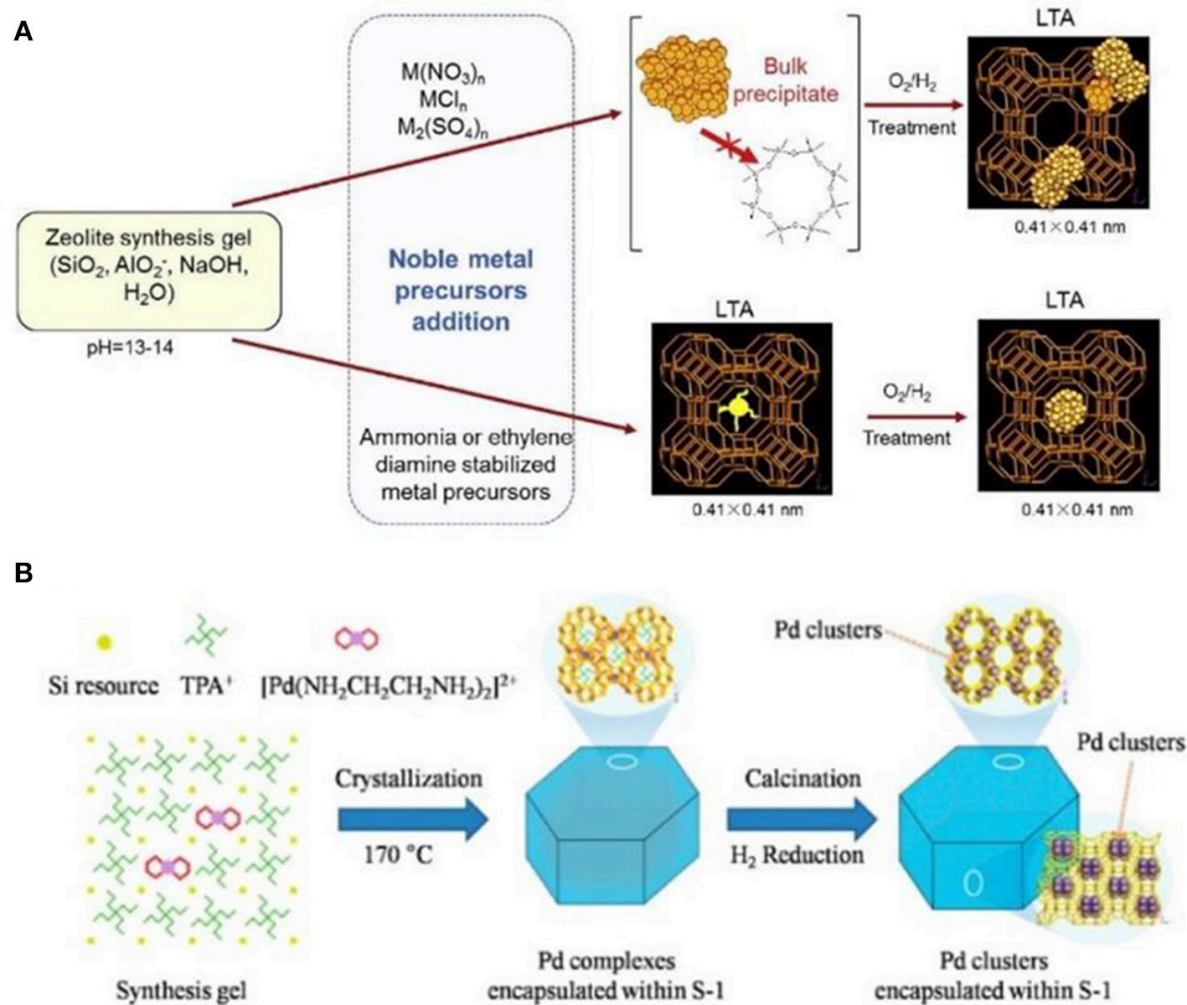
Schematic representation for the formation of zeolite-encapsulated metal NPs via one-pot hydrothermal synthesis process. **(A)** The synthesis process by using metal cations as the precursors (top) and the ligand-stabilized metal precursors (below) to produce LTA-encapsulated metal NPs. Reprinted with permission from Wu et al. (2014). Copyright 2014 Elsevier. **(B)** The MFI-encapsulated Pd NPs synthesized by using [Pd(NH_2_CH_2_CH_2_NH_2_)_2_]^2+^ as the Pd precursor. Reprinted with permission from Wang et al. (2016). Copyright 2016 American Chemical Society.

Comparatively speaking, metallic precursors are easy to be encapsulated inside the resultant zeolites. Wu et al. (2014) reported the encapsulation of noble metal NPs (Pt, Pd, Rh, Ir, Re, and Ag) in LTA voids (Figure 4A). If only metal cations were employed, premature precipitation of these metal hydroxides would be mainly formed. When some ligands (e.g., NH_3_, ethylenediamine) were used to stabilize metal cations and protect cationic moieties against precipitation, avoiding premature precipitation resulted in the zeolitic framework units to grow around the ligand-stabilized metal precursors. Finally, a series of metal NPs were successfully encapsulated within the small micropores of LTA zeolite. These composite materials exhibited excellent shape selectivity in both catalytic oxidative dehydrogenation of alkanols and hydrogenation of alkenes. Another important case by using this strategy was reported by Wang et al. (2016). The authors utilized organometallic complex ([Pd(NH_2_CH_2_CH_2_NH_2_)_2_]^2+^) as the Pd precursor to produce well-dispersed and ultrasmall Pd NPs in nanosized silicalite-1 (MFI). Pd NPs were successfully encapsulated within the intersectional channels of MFI with a diameter of ~1.8 nm (Figure 4B), which could be further tuned via the different synthetic conditions (using NaOH or KOH as the base source). They had also reported the preparation of subnanometric bimetallic NPs of Pd-M(OH)_2_ (M = Ni, Co) using the similar synthetic methods (Sun et al., 2017). These MFI-encapsulated metal NPs presented superior catalytic performance toward the dehydrogenation of formic acid.

From abovementioned cases, how to balance the crystallization growth of both zeolites and metal NPs is quite important for obtaining the well-dispersed and size-uniform metal NPs inside zeolite crystals. The type of metallic precursors and other synthetic conditions (e.g., alkalinity, silica sources) should be carefully considered. The corresponded catalytic activity may generally depend on how strong the confinement effect is between zeolite frameworks and metal NPs.

## Catalytic applications

**Figure 5:**
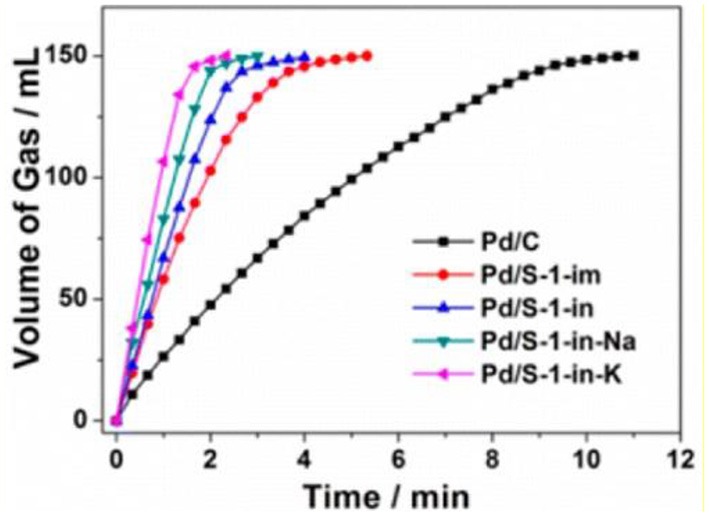
Volume of the generated gas (CO_2_ and H_2_) vs. time for the dehydrogenation of FA-SF over Pd/C and Pd/S-1. Reprinted with permission from Wang et al. (2016). Copyright 2016 American Chemical Society.

When metal NPs are encapsulated inside conventional zeolites, they are mainly confined in micropores and randomly distributed throughout the zeolite crystals. This type of catalysts proves to be appropriate for organic catalysis involving only small molecules. For example, when Pd cluster-containing zeolite catalysts (Pd/S-1, as shown in Figure 4B) was utilized to catalyse the dehydrogenation of formic acid and sodium formate (FA-SF) to produce H_2_ (Figure 5), Pd/S-1 exhibited complete decomposition of FA in a short time compared to the commercial Pd/C catalyst, also better than the Pd/S-1 catalyst obtained by the impregnation-prepared catalysts (Wang et al., 2016). However, although exhibiting excellent catalytic activity in the dehydrogenation process, this catalyst may miss their ability in organic reactions for the larger molecules, due to the limitation of small microporous channels or accessible void spaces. Additionally, even if the catalytic reactions involving organic molecules with small diameters (less than the micropores) happens in this type of composite catalysts, relatively slow diffusion in the interior zeolites would greatly influence the catalytic processes.

On the other hand, pure zeolite-encapsulated metal NP catalysts will be powerless to catalyse the reactions with big molecular volume (Ju et al., 2015; Dai et al., 2016; Kosinov et al., 2018). The difficulty for mass transfer and bulky molecule catalysis can be significantly improved via producing mesopores inside conventional zeolites as abovementioned. When immobilized on mesoporous zeolites, metal NPs can be not only distributed inside micropores, but also dispersed on the external surface of the mesopores. Those provide more catalytically active sites and easier ways to across the catalysts.

**Figure 6:**
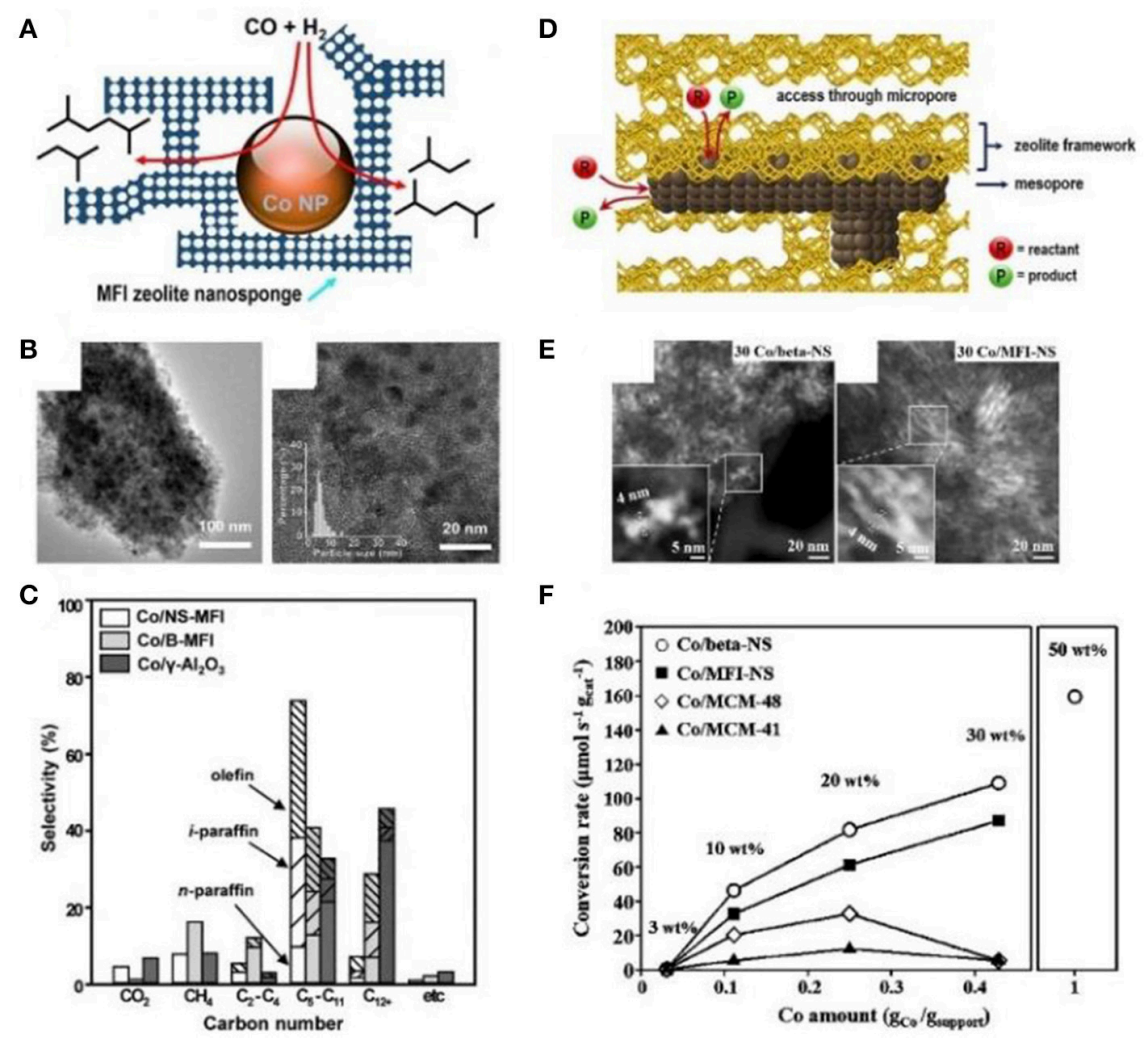
Mesoporous zeolite nanosponge-encapsulated Co NPs for Fischer-Tropsch reaction. **(A)** Schematic representation, **(B)** TEM images, and **(C)** product selectivity of mesoporous MFI nanosheets-encapsulated Co NPs catalysts. Reprinted with permission from Kim et al. (2014). Copyright 2014 American Chemical Society. **(D)** Schematic representation, **(E)** TEM images, and **(F)** CO conversion rates of mesoporous Beta and MFI nanosponge-encapsulated Co NPs with high loading amount, accessible through microporous windows at the encasing mesopore walls. Reprinted with permission from Han et al. (2018). Copyright 2017 American Chemical Society.

Han et al. (2018) and Kim et al. (2014) successfully prepared mesoporous zeolite nanosponge-encapsulated Co NPs as the efficient catalysts for Fischer-Tropsch (FT) synthesis. In the presence of amphiphilic surfactants containing multiquaternary ammoniums, zeolite nanosponge with ultrathin walls, which could be irregularly interconnected into three-dimensional mesoporous networks, had been synthesized. By means of the incipient wetness impregnation process using [Co(NO_3_)_2_·6H_2_O] as the metallic precursor, Co NPs were immobilized into the mesopores. Due to the narrow distribution of mesopore diameters, Co NPs possessed the uniform NP diameters of 4 nm (Figures 6A,B). Compared to bulk MFI zeolite or γ-Al_2_O_3_ supports, the cobalt-supporting zeolite nanosponges had higher catalytic performance in FT synthesis (Figure 6C). Owing to the strong confinement effect of mesopores, the catalyst exhibited high resistance to sintering, high conversion of CO, and long catalytic lifetime. Meantime, the thin MFI walls contributed to a high selectivity to branched hydrocarbons in the gasoline range (C_5_-C_11_). The authors further increased the loading amount of Co within zeolite Beta or MFI nanosponge, resulting in the construction of Co nanowires or networks along the mesoporous nanochannels (Figures 6D,E); (Han et al., 2018). Although the mesopores were blocked by Co nanostructures, the accessibility through microporous windows on the mesopore walls could also ensure a high catalytic activity toward FT synthesis (Figure 6F). Furthermore, the zeolite nanosponge-encapsulated Co nanostructures also exhibited high catalytic performance in benzene hydrogenation and furfural-to-γ-valerolactone conversion.

**Figure 7:**
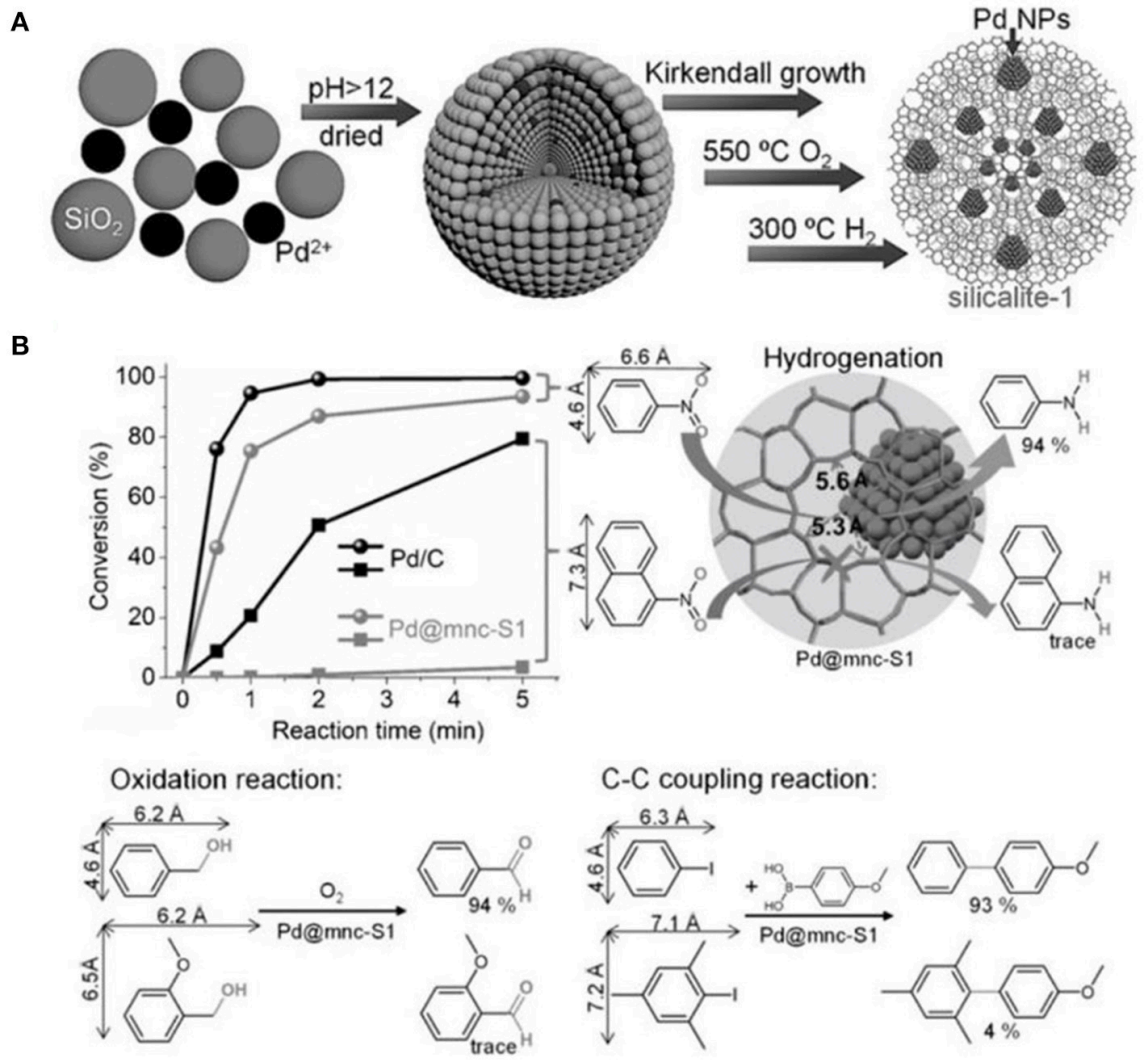
Mesoporous silicalite-1 nanocrystals-encapsulated Pd NPs (Pd@mnc-S1) with enhanced catalytic activity in various organic reactions. **(A)** The synthesis scheme for the preparation of Pd@mnc-S1 via *in situ* confinement process. **(B)** The different organic reactions (hydrogenation, oxidation, and C-C coupling reactions) catalyzed by Pd@mnc-S1. Reprinted with permission from Cui et al. (2016). Copyright 2016 Wiley.

Based on *in situ* confinement strategy, mesoporous silicalite-1 nanocrystals-encapsulated Pd NPs (Pd@mnc-S1) could be well-constructed by Cui et al. (2016) After mixing silica, Pd^2+^, small amount of polyvinyl pyrrolidone (PVP) and water, silicalite-1 nanocrystals with built-in mesopores were synthesized following the Kirkendall growth process (Figure 7A). The presence of PVP could prevent the aggregation of palladium oxide or hydroxide NPs under the high pH value. Metallic Pd NPs were obtained via successive calcination of the as-synthesized products in oxygen and hydrogen atmospheres. Pd NPs with a diameter in the range of 2~5 nm exhibited a high thermal and chemical stability, indicating the high encapsulation of Pd NPs inside the framework of silicalite-1 nanocrystals. The intrinsic micropores and Pd NPs in Pd@mnc-S1 resulted in good shape selectivity for a series of model reactions, such as hydrogenation, oxidation and C-C coupling reactions (Figure 7B). For example, catalytic hydrogenation conversion of nitrobenzene into aniline could be efficiently completed using Pd@mnc-S1 as the catalyst. However, only a negligible amount of 1-nitronaphthalene would be converted into naphthalen-1-amine over Pd@mnc-S1. As well-known, 1-nitronaphthalene possess the molecule size about 7.3 × 6.6 Å, bigger than the micropores of zeolite MFI (5.3 × 5.6 Å), resulting in the inability of Pd@mnc-S1 to catalyse the bulk organic molecules. The same results of shape selectivity had been found in the oxidation and C-C reactions. Additionally, the presence of mesopores inside zeolite supports greatly enhanced the mass-transfer efficiency and thus gave rise to the high catalytic activity. The authors further found that the number of acid sites in mesoporous H-ZSM-5 frameworks could play as “solid ligands” to activate the embedded Pd NPs for organic synthesis (e.g., Suzuki coupling reactions); (Ke et al., 2017).

**Figure 8:**
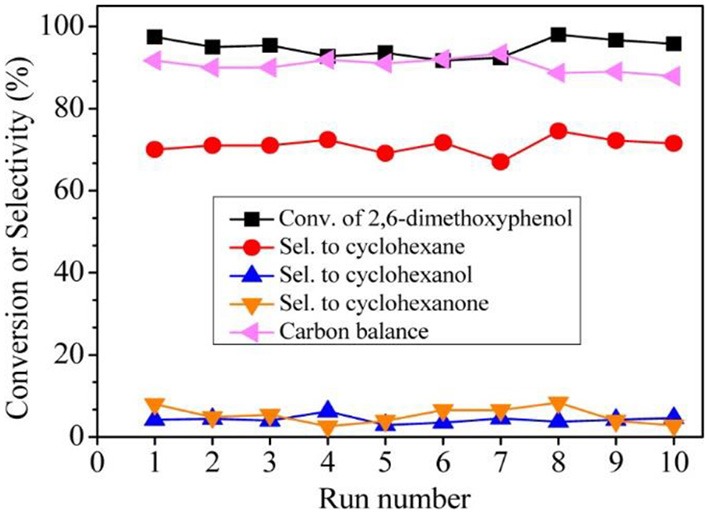
Recycling tests of catalyst Ru/HZSM-5-OM in the hydrodeoxygenation of 2,6-dimethoxyphenol. Reprinted with permission from Wang et al. (2015). Copyright 2015 American Chemical Society.

In order to catalyse the hydrodeoxygenation of the phenolic compounds in bio-oil to alkanes, Wang et al. (2015) immobilized Ru NPs into *b*-axis-aligned mesoporous ZSM-5 (Ru/HZSM-5-OM) as an efficient catalyst. Owing to the open mesopores and rich exposed acid sites, Ru/HZSM-5-OM exhibited better catalytic activity in the hydrodeoxygenation of bulky 2, 6-dimethoxyphenol (as a model reaction) than bulk or mesoporous HZSM-5-encapsulated Ru NPs. This is because the bulky reactant molecule could easily transfer through the open mesopores and complete the reaction on the external/mesopore acid sites in HZSM-5-OM. A high conversion rate and cyclohexane selectivity could be reached when using Ru/HZSM-5-OM as the catalyst. Ru/HZSM-5-OM also presented excellent recyclability, keeping the high conversion (95.8%) and cyclohexane selectivity (69.0%) after recycling ten times (Figure 8). Due to the fine confinement effect from HZSM-5-OM, Ru NPs could be well-stabilized in zeolitic framework as no Ru species in the liquid had been detected under the reaction conditions or after the recycling test.

**Figure 9:**
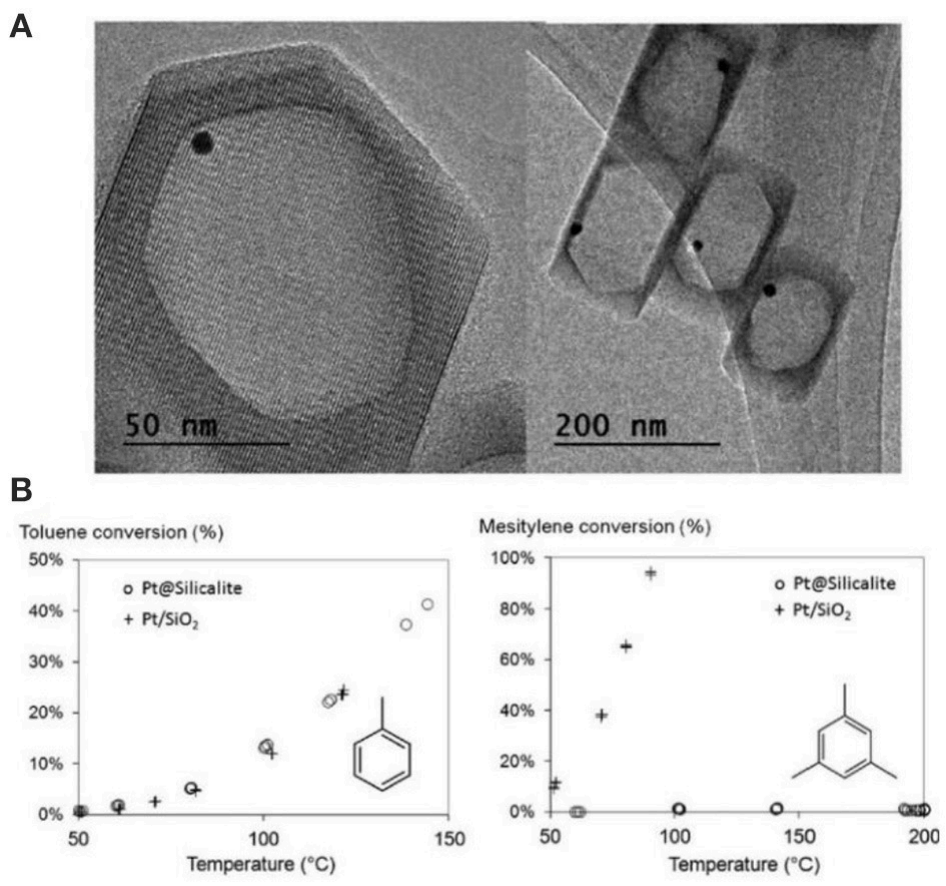
Hollow silicalite-1-encapsulated Pt NPs (Pt@Sil-1) as size selective hydrogenation of aromatic compounds. **(A)** TEM images of Pt@Sil-1. **(B)** Conversion vs. temperature plots for the hydrogenation of toluene and mesitylene from Pt@Sil-1 and Pt/SiO_2_ catalysts. Reprinted with permission from Li et al. (2014b). Copyright 2014 Royal Society of Chemistry.

Another interesting strategy to build porous zeolite-encapsulated metal NP catalysts is promised to utilize hollow zeolites as the inorganic supports (Dong et al., 2003; Ren et al., 2007; Li et al., 2014b,c; Pagis et al., 2016; Prieto et al., 2016). The structural features endow them into hierarchically macro-microporous or macro-meso-microporous systems, both of which are structurally beneficial for mass transfer of the reactants and products. The active sites of metal NPs on relatively thin shell are easily accessible. For example, Li et al. (2014b) produced Pt NPs inside hollow silicalite-1 crystals as the composite catalysts for size-selective hydrogenation of aromatic compounds (Figure 9A). The hydrogenation product of methylcyclohexane from toluene possessed a kinetic diameter less than MFI micropores and thus easily diffused through the zeolitic shell. Contrastively, the product of trimethylcyclohexane from mesitylene could not go across the zeolite shell to reach the reaction sites on Pd NPs, resulting in the poor catalytic activity of Pt@Sil-1 on the hydrogenation of bulky mesitylene (Figure 9B). Although the catalyst in this case did not perform the activity toward bulky molecules (only size selectivity), it reminds us that, if some mesopores are created on the zeolite shell (hollow mesoporous zeolites), corresponded catalytic performance will be greatly improved in both shape selectivity and bulky molecular catalysis. Owing to the presence of hierarchically macro-meso-micropores in one zeolite support, the composite catalysts after the encapsulation of metal NPs will be most efficient for various organic reactions.

As for the most cases mentioned above, the mesopores in the final catalysts are not particularly ordered, which may lead to the weak mass transfer efficiency compared to the ordered ones. How to construct a set of ordered mesopores inside the zeolites supports for the encapsulation of metal NPs is always a big challenging subject. The catalytic performances (e.g., conversion, shape selectivity, stability) in these expected catalysts should be greatly improved. The catalysts are theoretically employed in many fields of organic or small inorganic molecule reactions.

## Summary and perspectives

Mesoporous zeolite is a type of ideal inorganic supports for the encapsulation of metal NPs to produce composite catalysts for various organic reactions. In this review, the advantages of mesoporous zeolites as the supports are presented in detail with the comparison to other nanomaterials like metal or non-metallic oxides, carbon, polymers, and porous frameworks. The main strategies for the construction of mesoporous zeolites and the encapsulation methods for metal NPs have been summarized as these two aspects are extremely important to combine mesoporous zeolites and metal NPs into composite catalysts. Several representative examples exhibited well-catalytic performances in reactant conversions, shape selectivities, and stabilities in many organic catalytic reactions. There are still a lot of challenges in the preparation of mesoporous zeolite-encapsulated metal NPs, such as the degree of mesoporous order, the dispersity and size distribution of encapsulated metal NPs, and the large-scale synthesis, and so on. Additionally, the structure-performance relationship between catalytic reactions and catalyst structures (different mesopores, micropores, and metals) should be carefully revealed in the future.

As for the construction of ordered mesoporous zeolite-encapsulated metal NP catalysts, some purposed strategies are presented. The appropriate process should be the one-pot synthesis via the mixing of zeolite gel and optimized metallic precursors (Wu et al., 2014) under the presence of some special structural-directing surfactants (Na et al., 2011) or ordered hard templates (Chen et al., 2011). The most metal precursors can be retained after the removal of templates and reduced *in situ* into NPs inside the micropores. In this way, the ordered mesopores can run throughout zeolite crystals and meanwhile metal NPs are tightly immobilized inside microporous frameworks, resulting in the higher mass transfer efficiency and enhanced catalytic activity, especially for the bulky molecular catalysis. We hope this review can provide useful insights on the synthesis of zeolites supported metal NP composite catalysts and the understanding of the relationship between the catalytic performance and nanostructure of composite catalysts.

## Author contributions

All authors listed have made a substantial, direct and intellectual contribution to the work, and approved it for publication.

### Conflict of interest statement

The authors declare that the research was conducted in the absence of any commercial or financial relationships that could be construed as a potential conflict of interest. The handling editor declared a past supervisory role with one of the authors BL.
